# Establishment of the First Comprehensive Adult and Pediatric Hematopoietic Stem Cell Transplant Unit in the United Arab Emirates: Rising to the Challenge

**DOI:** 10.3390/clinpract12010010

**Published:** 2022-01-28

**Authors:** Humaid O. Al-Shamsi, Amin Abyad, Panayotis Kaloyannidis, Amro El-Saddik, Ahmad Alrustamani, Ibrahim Abu Gheida, Azzam Ziade, Norbert W. Dreier, Urfan Ul-Haq, Thanda Lucy Ann Joshua, Abdul Rahman El Kinge, Ritika Coelho, Dima Ibrahim, Mehdi Afrit, Bilal Al-Lababidi, Zainul Aabideen, Mayur Sabhani, Rakeshkumar Shah, Ghaith Makhlouf, Lana Iskandaerani, Faryal Iqbal, Shiny Narayanan, Mohammed Ameen, Theresa Morrison, Charbel Khalil, Kayane Mheidly

**Affiliations:** 1 Department of Oncology and Innovation and Research Center, Burjeel Cancer Institute, Burjeel Medical City, Abu Dhabi, United Arab Emirates; amin.abyad@burjeelmedicalcity.com (A.A.); amro@vpshealth.com (A.E.-S.); ahmed.alrustamani@burjeelmedicalcity.com (A.A.); ibrahimabugheida@burjeelmedicalcity.com (I.A.G.); azzamziade1@icloud.com (A.Z.); norbert.dreier@burjeel.com (N.W.D.); urfan.haq@burjeel.com (U.U.-H.); thanda.joshua@burjeel.com (T.L.A.J.); ritika.coelho@burjeelmedicalcity.com (R.C.); Dimaibrahim90@hotmail.com (D.I.); mehdi.afrit@burjeel.com (M.A.); dr.bilalbak@burjeel.com (B.A.-L.); zainul.aabideen@burjeelmedicalcity.com (Z.A.); mayur.sabhani@vpshealth.com (M.S.); rakeshkumar.shah@burjeelmedicalcity.com (R.S.); ghaith.makhlouf@burjeelmedicalcity.com (G.M.); lana.iskandaerani@burjeelmedicalcity.com (L.I.); faryal.iqbal@burjeelmedicalcity.com (F.I.); shiny.narayanan@burjeelmedicalcity.com (S.N.); mohammed.ameen@burjeel.com (M.A.); theresa.morrison@burjeelmedicalcity.com (T.M.); charbelk3@hotmail.com (C.K.); kayane.mheidly@burjeelmedicalcity.com (K.M.); 2 Emirates Oncology Society, Dubai, United Arab Emirates; 3 College of Medicine, University of Sharjah, Sharjah, United Arab Emirates; 4 Adult Hematology and SCT Department, King Fahad Specialist Hospital, Dammam 32253, Saudi Arabia; pkaloyannidis@yahoo.gr; 5 NMC Royal Hospital, Sharjah, United Arab Emirates; ardkinge@gmail.com

**Keywords:** bone marrow transplant, stem cells, apheresis, middle east, gulf

## Abstract

Hematopoietic stem cell transplantation (HSCT) is increasingly indicated for various malignant and non-malignant diseases. In the United Arab Emirates (UAE), patients that could benefit from the procedure commonly need to seek medical care abroad in view of the lack of a comprehensive HSCT facility that could offer the full spectrum of interventions and monitoring protocols. This comes with considerable challenges related to coverage and logistics of travel. It also limits the continuity of clinical care, and presents inconvenience to patients who come from a different cultural background. In this article, we share our experiences and lessons learned during the establishment of the first comprehensive adult and pediatric HSCT unit in the UAE that is designed to cater for local citizens and residents, as well as neighboring countries facing similar availability challenges.

## 1. Introduction and Background

Hematopoietic stem cell transplantation (HSCT) has become a well-established, lifesaving or curative treatment option for various malignant and non-malignant hematologic diseases, solid tumors, and immune disorders [1]. It has gradually evolved from being considered an “ultima ratio” option to being integrated as an essential part of international treatment guidelines. Recommendation usually follows careful risk assessment to weigh the risk associated with the disease and non-transplant strategies versus that of the transplantation procedure, which relies on the stage and duration of disease, individual patient profile and comorbidities, transplant protocol, conditioning regimen, donor type, and stem cell source [1,2]. Experience and expertise in HSCT are essential to the application of novel cell and gene therapies, which have the potential to reshape the therapeutic options for various diseases [3,4,5]. The success of HSCT programs is tightly linked to the contemporary design and establishment of a comprehensive and dedicated unit with multidisciplinary teams that follow international standards. In this work, we describe our journey through establishing the first comprehensive adult and pediatric HSCT unit in the United Arab Emirates (UAE), and share our strategy and lessons learned to serve as insights for colleagues from countries with similar ongoing plans. 

## 2. Opportunity Assessment and Addressing a Critical Unmet Need

The UAE is comparatively a young and new state, established in December 1971 as a federation of seven Emirates. It has one of the world’s fastest-growing economies, fueled by visionary planning, investments, and an unparalleled infrastructure. Notably, the healthcare sector has witnessed a swift evolution over the past few decades, with the establishment of various state-of-the-art healthcare facilities with international collaboration. Nonetheless, a considerable number of patients continue to seek medical care abroad, especially for cancer treatment [6]. Around 8000 Emirati patients have received some form of medical care abroad in the last few years for malignant and non-malignant conditions, funded by the government [7]. The same largely applies for expatriate patients, who often elect to return home for medical treatment for various logistic and economic reasons. 

When it comes to HSCT, seeking medical care abroad becomes inevitable in view of the lack of comprehensive HSCT units that cater to the local demand. Based on data from the Emirate of Abu Dhabi Department of Health pertaining to Emirati patients, a total of 161 adult and 164 pediatric patients underwent HSCT outside the UAE between the years 2016–2018. This not only comes at a high expenditure, with coverage and logistic hurdles for a procedure that requires strict and dedicated peri-procedural management, but also impacts the psychological wellbeing of the patients, and disrupts the continuity of care. Returning patients often come with suboptimal understanding of their medical care that was provided abroad, as well as missing medical documentation and reports, which might put them at disadvantages of treatment interruption or variation in treatment approaches between medical teams locally and abroad, if there are communication gaps [8]. Patient experiences are also often compromised by challenges of language differences, cultural discrepancies, and ethical and religious issues that could impact their overall quality of care. Today, medical tourism is further complicated by limited mobility and COVID-19 infection hazard during travel, which mandates strict travel arrangements, and is even more worrisome in an already immunocompromised patient population. Thus, with the ongoing pandemic and restrictions in travel, the need for national and self-sustained medical services including HSCT becomes essential [8].

The epidemiology of malignant and non-malignant hematologic indications in the UAE further calls for local solutions, such as HSCT. Standardized collection of cancer incidence started around a decade ago, when the Ministry of Health and Prevention (MOHAP) established the UAE National Cancer Registry (UAE-NCR) with the aim to access medical information while protecting the data confidentiality of the patients, entitled the “UAE National Cancer Registry”. This is a population-based cancer registry, and includes both local Emirati patients (UAE citizens) and expatriate residents (non-UAE citizens) [9]. The latest available annual report form the UAE-NCR relates to incident cases during the year 2017, which included 314 (223 expatriate, 91 Emirati) cases of leukemia, 50 (35 expatriate, 15 Emirati) cases of multiple myeloma, 172 (126 expatriate, 46 Emirati) cases of non-Hodgkin’s lymphoma, and 75 (52 expatriate, 23 Emirati) cases of Hodgkin’s lymphoma [9]. When it comes to non-malignant indications, the UAE has considerably high rates of several heritable hematologic disorders that have been historically endemic to the region; some that come with significant public health burden and healthcare utilization, such as thalassemia [10]. 

Taken together, this allowed us to recognize a high unmet need for the establishment of a local adult and pediatric comprehensive HSCT unit that can service a large number of patients who can benefit from the procedure “at home” with all the clinical, logistic, and economic advantages this entails. This would also allow patients from neighboring countries with similar challenges to seek HSCT in an easily accessible neighboring country that has always opened its door for foreigners, and would facilitate their transition and integration. Of note, the only HSCT service that has been available in the UAE since 2019 comes through a private provider, and until October 2021, to the best of the authors’ knowledge, they have completed ten noncryopreserved autologous HSCT cases. 

## 3. Conceptualization and Journey through Establishment

Our aim was to establish a comprehensive HSCT unit with international standards based on the European Society for Blood and Marrow Transplantation (EBMT) recommendations, in support of the Department of Health’s and UAE government’s vision to provide the best care for UAE citizens and residents, and to make Abu Dhabi an emerging hub for global medical tourism.

Our HSCT unit was established as part of our comprehensive cancer center (Burjeel Cancer Institute) in Burjeel Medical City, incepted in 2015 as the flagship medical care facility of VPS Healthcare, spanning 1.1 million square feet in the capital, Abu Dhabi. Over the last year, our oncology service was able to transform into a state-of-the-art facility that offers multidisciplinary cancer care, including medical and surgical oncology, radiation oncology, nuclear medicine, and palliative care through a dedicated team of experts with international training. It is the first and only European Society of Medical Oncology (ESMO) designated center of integrated oncology and palliative care in the UAE. 

Our journey with HSCT started in February 2021 with the formation of a taskforce which consisted of clinical adult and pediatric hematologists with extensive HSCT experience, a chief nursing officer, a human resources representative, oncology and hematology pharmacists, adult and pediatric infectious disease specialists, a laboratory director, a quality director, and international advisors with experience in establishing HSCT services from Saudi Arabia and India [11]. We started by assessing the current infrastructure and existing manpower to identify resource needs. Our first step was to top up our manpower with any missing functions (as detailed below) to ensure we get the proper “buy in” from those who would be directly involved in the procedure. 

**Figure 1:**
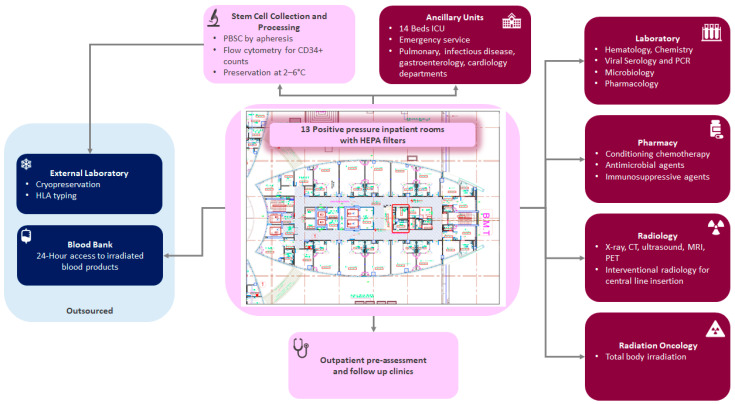
Hematopoietic stem cell transplant unit current set up. HLA, human leukocyte antigen; PCR, polymerase chain reaction; HEPA, high-efficiency particulate absorbing; PBSC, peripheral blood stem cells; ICU, intensive care unit; CT, computed tomography; MRI, magnetic resonance imaging; PET, positron emission tomography.

A dedicated positive pressure unit with 13 inpatient rooms with high-efficiency particulate absorbing (HEPA) filters was purposely built (Figure 1). Adult and pediatric HSCT nurse specialists with extensive experience in chemotherapy administration, infection control, and handling of stem cell products were recruited to manage the unit. Outpatient examination rooms were also set up for pretransplant evaluation and consultations, consenting, patient education, and follow-up with a dedicated HSCT coordinator. Clinical care pathways were then established with ancillary medical units, including a full-capacity 14 beds intensive care unit, an active emergency room, as well as gastroenterology, pulmonary, cardiology, and infectious disease departments. Dedicated ambassadors from such units were assigned to align the services with the acute and chronic care needs of HSCT patients. Considering the peculiarities of infectious disease control in an HSCT setting, a US-trained solid and HSCT infectious disease specialist was also recruited. 

Stem cell collection was made feasible though a new apheresis machine placed in a dedicated collection room. Peripheral blood stem cell apheresis capability was thus established, with a clinical scientist specialized in stem cell therapy, and dedicated for stem cell apheresis and processing. CD34+ cell count is done on site by flow cytometry, and collected cells are preserved in our facility in a specific fridge at a temperature between 2 and 6 °C. A cellular therapy unit is currently being established for cryopreservation and stem cell processing. Until then, cryopreservation of stem cell products is outsourced to an external laboratory. The HSCT program fulfilled the required criteria for establishing HSCT programs as recommended by the Worldwide Network for Blood and Marrow Transplantation (WBMT) workshop [12].

Rules in the UAE do not allow having hospital-based blood banks; hence, a dedicated taskforce visited the central Abu Dhabi blood bank and outlined our need and plan for the HSCT service, and communication was established to ensure 24-h access to blood products irradiated for patients who undergo HSCT. We have close communication with the blood bank for pre-planning, and we also keep our own reserves for emergency use. 

Our center already had a state-of-the-art automated laboratory for routine hematology and chemistry testing, and the capacity for viral serology and polymerase chain reaction (PCR) testing and microbiology for common bacterial and fungal cultures. The laboratory also secured outsourced access to pharmacologic assays of drug levels. Our pharmacy protocols were also revisited to ensure conditioning chemotherapy, antimicrobial agents, and immunosuppressive agents were readily procured. Our radiology department was made aware of our various support needs, including ultrasounds, X-rays, computed tomography, magnetic resonance imaging, and positron emission tomography scans. Central venous catheters placements are also available through the interventional radiology team. A total body irradiation protocol was also formulated by our radiation oncology department in case it was needed.

The overall function of the unit is governed through written institutional protocols, with standard operating procedures and guidelines. Regular audits of various HSCT procedures and patient treatment outcomes are conducted on a daily basis through a round done by the HSCT specialist, and once weekly by with the interdisciplinary team (including the infectious disease specialist, nurses, nutritionist, psychologist, and any other specialist needed for the specific case). The unit received a Department of Health approval with a 100% score ranking in August 2021. 

## 4. Our First Cases

We focused, as a starting point, on autologous HSCT for multiple myeloma, given that it represents the most common transplant indication worldwide, and is a relatively simple protocol for medical staff adaptability and training purposes. We first evaluated eight cases, and our approach was to select low-risk HSCT candidates. Two cases were selected to be conducted during the same week in October 2021. Our first patient was a 46-year-old male who was diagnosed with multiple myeloma more than 15 years ago. He was initially treated using a conventional chemotherapy-based protocol, VAD (vincristine, doxorubicin, and dexamethasone), and attained remission, but was not offered frontline HSCT due to non-availability of the service at that time, and the financial incapacity of the patient to travel abroad for the procedure. Recently, the patient had documented relapse, and achieved partial response with a bortezomib, lenalidomide, and dexamethasone (VRd) regimen. Patient eligibility testing showed he was in partial response as per the International Myeloma Working Group criteria, enabling him to be a good candidate for intensification treatment by autologous HSCT. However, the challenge was not only limited to eligibility testing, but also included clearance from various aspects of his disease. Although the patient did not have any serious comorbidities, his medical history was positive for numerous prior infections. To ensure patient safety, he went into extensive investigations for possible latent infections (including a bronchoscopy). After all infection prevention precautions, and after very thorough discussions with staff from various medical specialties, including cardiology, pulmonary, and infectious disease, the patient was cleared, and a peri-transplant antimicrobial prophylaxis program was scheduled. The patient received a noncryopreserved autologous HSCT with melphalan conditioning. 

The second patient was a 53-year-old male referred to our facility from Lebanon after he was recently diagnosed with high-risk multiple myeloma. He was treated with daratumumab and VRd, and was planned for autologous HSCT after first remission. Unfortunately, due to the financial crisis occurring in Lebanon, the patient was no longer able to receive autologous transplant as an intensification treatment [13,14]. The case was discussed and presented to our senior management, and a decision was made to cover the patient’s full treatment and accommodation. The patient arrived in UAE shortly after, started eligibility testing, and was found to be in complete remission. He received a noncryopreserved autologous HSCT with melphalan conditioning.

## 5. Outreach Activity and Building Community Trust

We have initiated various campaigns in collaboration with designated medical societies, and local print and digital media, to increase public awareness on cancer (including hematologic malignancies) screening, diagnosis, and treatment. The aim of such campaigns was also to make the public aware of advances in care, and to highlight that they are now increasingly available locally. We have also announced our HSCT service through direct communication with hematologist and oncologist networks across the UAE and the region, and facilitated referral and transfer pathways. Moreover, we have established the first comprehensive UAE-wide HSCT weekly virtual multidisciplinary team (MDT) meeting to discuss cases, and share experiences and expertise. We have also set up a weekly MDT with experts from Lebanon who have long-standing experience with HSCT for idea sharing and case reviews. 

## 6. Future Directions

**Figure 2:**
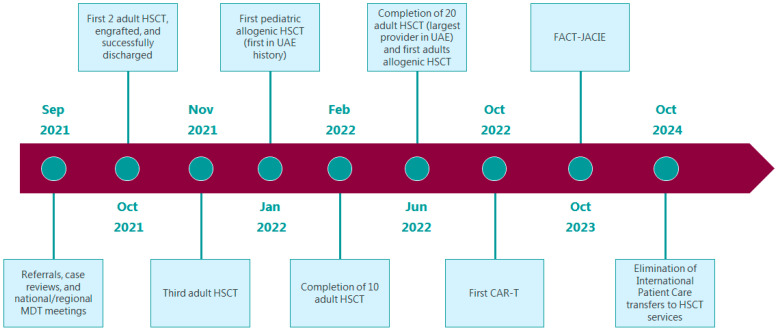
Hematopoietic stem cell transplant unit future plans. HSCT, hematopoietic stemcell transplantation; CAR-T, Chimeric antigen receptor T; FACT-JACIE, Foundation for the Accreditation of Cellular Therapy–Joint Accreditation Committee.

The HSCT unit is expected to complete 10 cases by February 2022, aiming to be the largest HSCT service provider in the UAE by June 2022 by completing 20 cases by then. Our target is to maintain volumes of greater than or equal to 10 new patients/year for autologous transplantation, and 10 new patients/year for allogeneic transplantation. The HSCT team is also preparing for the first pediatric allogenic transplant in the UAE’s history by January 2022. We are also preparing for the first adult allogenic transplantation by June 2022. Additionally, we are targeting October 2022 for our first Chimeric antigen receptor T (CAR-T) cell therapy. By October 2023, we aim for the HSCT unit to be accredited by the Foundation for the Accreditation of Cellular Therapy—Joint Accreditation Committee (FACT-JACIE). By October 2024, we aim for the complete elimination of international patient care transfers related to HSCT services outside the UAE (Figure 2).

## 7. Conclusions

As cliché as this may sound, our experience made us realize that where there is a will, there is a way. We have learned through this experience how critical change management and stakeholders’ alignment are for specialized services’ successful establishment, and how they could delay or accelerate the execution of such a program. Initially, we faced challenges dealing with intrinsic resistance and motivating local teams; this was slowly resolved through creating a culture for the change required through defining the patients’ needs, and the team’s ambitious goal. We focused on improving the communication platform, and celebrating success at every stage of the process. We also held a number of learned sessions with the core team to optimize the establishment process. We fully recognize that there is a long road ahead, and there is still much to do to grow our unit in terms of full-spectrum services and the patient pool, but we remain confident that the local and international collaborations we are actively establishing will support our transition to become a center of excellence for cancer care and research. Our ethics and standards are based on international and most-updated guidelines, which will allow us to have the trust of the local community and neighboring countries.

## Data Availability

No new data were created or analyzed in this study. Data sharing is not applicable to this article.

## References

[1] Duarte R.F., Labopin M., Bader P., Basak G.W., Bonini C., Chabannon C., Corbacioglu S., Dreger P., Dufour C., Gennery A.R. (2019). Indications for haematopoietic stem cell transplantation for haematological diseases, solid tumours and immune disorders: Current practice in Europe, 2019. Bone Marrow Transpl..

[2] Watts M.J., Linch D.C. (2016). Optimisation and quality control of cell processing for autologous stem cell transplantation. Br. J. Haematol..

[3] Sagoo P., Gaspar H.B. (2021). The transformative potential of HSC gene therapy as a genetic medicine. Gene Ther..

[4] Huang R., Li X., He Y., Zhu W., Gao L., Liu Y., Gao L., Wen Q., Zhong J.F., Zhang C. (2020). Recent advances in CAR-T cell engineering. J. Hematol. Oncol..

[5] Elverum K., Whitman M. (2020). Delivering cellular and gene therapies to patients: Solutions for realizing the potential of the next generation of medicine. Gene Ther..

[6] Al-Shamsi H.O., Al-Hajeili M., Alrawi S. (2018). Chasing the Cure around the Globe: Medical Tourism for Cancer Care from Developing Countries. J. Glob. Oncol..

[7] (2014). Itihad: 600 Million Dirhams Annually for Cancer Patient Treatment Abroad. http://www.alittihad.ae/details.php?id=90507&y=2014.

[8] Al-Shamsi H.O., Abu-Gheida I., Rana S.K., Nijhawan N., Abdulsamad A.S., Alrawi S., Abuhaleeqa M., Almansoori T.M., Alkasab T., Aleassa E.M. (2020). Challenges for cancer patients returning home during SARS-CoV-19 pandemic after medical tourism—A consensus report by the emirates oncology task force. BMC Cancer.

[9] Ministry of Health & Prevention (2017). Cancer Incidence in United Arab Emirates Annual Report of the UAE-National Cancer Registry-2017.

[10] Abu-Shaheen A., Heena H., Nofal A., Abdelmoety D.A., Almatary A., Alsheef M., AlFayyad I. (2020). Epidemiology of Thalassemia in Gulf Cooperation Council Countries: A Systematic Review. BioMed Res. Int..

[11] Al-Hashmi H., Alsagheir A., Estanislao A., Bacal J., Alsuhebah A., Alblowe B., Raslan H., Alsaber A., Albahrani A., Almulhem N. (2020). Establishing hematopoietic stem cell transplant programs; overcoming cost through collaboration. Bone Marrow Transpl..

[12] Pasquini M.C., Srivastava A., Ahmed S.O., Aljurf M., Atsuta Y., Doleysh C., Galeano S., Gluckman E., Greinix H., Hale G.A. (2019). Worldwide Network for Blood and Marrow Transplantation Recommendations for Establishing a Hematopoietic Cell Transplantation Program, Part I: Minimum Requirements and Beyond. Biol. Blood Marrow Transpl..

[13] Devi S. (2020). Economic crisis hits Lebanese health care. Lancet.

[14] Shallal A., Lahoud C., Zervos M., Matar M. (2021). Lebanon is losing its front line. J. Glob. Health.

